# Dengue epidemic in southern Vietnam, 1998.

**DOI:** 10.3201/eid0604.000421

**Published:** 2000

**Authors:** D Q Ha, N T Tien, V T Huong, H T Loan, C M Thang

**Affiliations:** Pasteur Institute, Ho Chi Minh City, Vietnam. pvtu@netnam2.org.vn

## Abstract

A widespread epidemic of dengue hemorrhagic fever (DHF) occurred in southern Vietnam in 1998, with 438.98 cases/100,000 population and 342 deaths. The number of DHF cases and deaths per 100,000 population increased 152.4% and 151.8%, respectively, over a 1997 epidemic. Dengue viruses were isolated from 143 patient blood samples; DEN-3 virus was identified as the predominant serotype, although a resurgence of DEN-4 was noted.


### Dengue Epidemic in Southern Vietnam, 1998

**Do Quang Ha, Nguyen Thi Kim Tien, Vu Thi Que Huong, Huynh Thi Kim Loan, Cao Minh Thang**
Pasteur Institute, Ho Chi Minh City, Vietnam

A widespread epidemic of dengue hemorrhagic fever (DHF) occurred in southern Vietnam in 1998, with 438.98 cases/100,000 population and 342 deaths. The number of DHF cases and deaths per 100,000 population increased 152.4% and 151.8% , respectively, over a 1997 epidemic. Dengue viruses were isolated from 143 patient blood samples; DEN-3 virus was identified as the predominant serotype, although a resurgence of DEN-4 was noted.

Since 1963, the incidence of dengue hemorrhagic fever (DHF), a leading cause of hospitalization and death in children, has steadily increased in Vietnam. In 1998, a widespread DHF epidemic affected 19 provinces in southern Vietnam (Figure 1); 119,429 cases of DHF and 342 deaths were reported (Figure 2); and the rates per 100,000 population were 438.98 and 1.26, respectively, for a case-fatality rate of 0.29%, an increase of 152.4% and 151.8%, respectively, over those of a 1997 epidemic (288.02 and 0.83)(1). The epidemic curve was similar to those of previous years: cases increased substantially from June to November (1-4). Peak transmission occurred from July to September, closely associated with the rainy season, a breeding period for the mosquito vector. DHF cases were reported in the first quarter in Ben Tre (1,387.2/2.4/100,000), Binh Phuoc (635.1/0), and Kien Giang provinces (568.4/2.9).

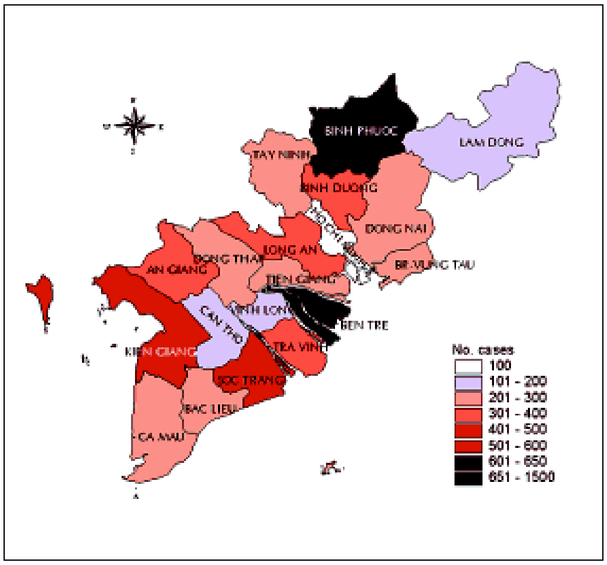

Figure 1. Nineteen provinces in southern Vietnam with mortality rates per 100,000 population, 1998.

We describe epidemiologic, virologic, and serologic studies carried out during the epidemic.

### The Study

Reports of DHF cases and deaths were gathered by hospitals and Departments of

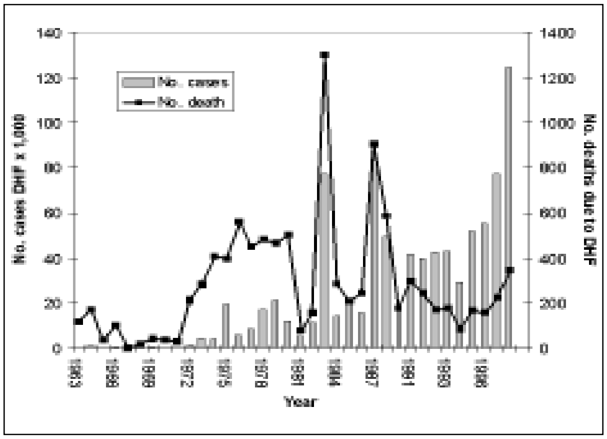

Figure 2. Reported cases of dengue hemorrhagic fever in southern Vietnam, 1963-1998.

Address for correspondence: Do Quang Ha, Pasteur Institute, 167 Pasteur Street, District 3 Ho Chi Minh City, Vietnam; fax: 84-8-8231419; e-mail:pvtu@netnam2.org.vn.

Hygiene and Preventive Medicine at the district level, then sent to the Provincial Centers of Preventive Medicine. These data were reported weekly to the Pasteur Institute in Ho Chi Minh City. Seventeen of the 19 provinces submitted blood samples to the Institute for virus isolation. One hundred forty-three dengue viruses were isolated from 1,236 blood samples, for a positivity rate of 11.6% (Table 1). Although DEN-1 and DEN-2 had been the most common serotypes (1-4), DEN-3 was isolated in 15 provinces.

The blood samples were obtained on days 1 to 4 after the onset of illness and were stored at -20°C or -70°C before being injected into C6/36 (*Aedes albopictus*) cell cultures seeded at 3 x $10^5$ cells per mL in 1-mL glass tubes. Undiluted blood was injected into duplicate tubes (0.05 mL per tube) and incubated at 28°C for 7 days. Infected cell cultures were harvested and assayed for dengue virus by the direct and indirect fluorescent antibody techniques, with the monoclonal antibody SLE 6B6C-1/FITC conjugate and four serotype-specific monoclonal antibodies: DEN-1 (Hawaii 15F3-1-15 and D2-1F1-3), DEN-2 (NGC 3H5-1-21), DEN-3 (H87 5D4-11-24), DEN-4 (H241 1H10-6-7), and Japanese encephalitis (Nakayama 14H5) (5). To detect dengue-specific IgM antibody, samples were tested by IgM-capture enzyme-linked immunosorbent assay (Mac-ELISA) by using the monoclonal antibody SLE 6B6C-1/HRP conjugate (6).

Sixteen of 19 provinces in southern Vietnam submitted patient sera for dengue serodiagnosis. Seropositive results were seen in all provinces throughout the year, and the confirmation rates increased during the DHF season (Table 2). Despite the high sensitivity and specificity of Mac-ELISA for dengue diagnosis, the seropositivity rates in eight provinces were low (< 50%). Clinical diagnoses of DHF during the epidemic in these provinces may have been overestimated, especially in cases of suspected DHF or fever of

Table 1. Dengue viruses isolated, by province, 1998

| No. | Prov. | Jan | Feb | Mar | Apr | May | Jun | Jul | Aug | Sep | Oct | Nov | Dec | Total |
|---|---|---|---|---|---|---|---|---|---|---|---|---|---|---|
| 1 | Lam Dong | | | | 2D3 | | 2D3 | 1D3 | 5D1 | | | | | 5D1 |
| | | | | | | | | | 5D3 | | | | | 10D3 |
| 2 | Dong Nai | | | | | | 1D2 | 1D2 | | | 1D2 | | | 3D2 |
| | | | | | | | | 1D3 | | | 3D3 | 1D3 | | 5D3 |
| 3 | Binh Phuoc | | | | | | 1D3 | | | | | | | 1D3 |
| 4 | Binh Duong | | | | | | | | | | | 2D3 | | 2D3 |
| 5 | BR-V Tau | | | | | | 1D1 | | | | | | | 1D1 |
| 6 | HCMC | 2D3 | 1D3 | | | | 2D3 | 2D3 | 2D3 | 2D3 | 7D3 | | | 18D3 |
| 7 | Tien Giang | | | | | | 1D1 | 1D1 | 4D3 | | 3D2 | | | 2D1 |
| | | | | | | | 5D3 | 1D2 | | | 1D3 | | | 4D2 |
| | | | | | | | | | | | | | | 21D3 |
| 8 | Dong Thap | 1D4 | | | | | | | | | | | | 1D4 |
| 9 | Vinh Long | | 2D1 | | | | 1D2 | | | | | | | 2D1 |
| | | | 6D2 | | | | | | | | | | | 7D2 |
| | | | 3D3 | | | | | | | | | | | 3D3 |
| 10 | Tra Vinh | | | | | | | 1D1 | | | 4D3 | 2D4 | | 1D1 |
| | | | | | | | | 4D2 | | | 1D4 | | | 4D2 |
| | | | | | | | | 1D3 | | | | | | 5D3 |
| 11 | Can Tho | | | | | 1D3 | | | 1D3 | | | | | 2D3 |
| 12 | Soc Trang | | | | | | | | 1D1 | | | | | 1D1 |
| | | | | | | | | | 5D3 | | | | | 5D3 |
| 13 | Ben Tre | 1D1 | 1D1 | | | 2D3 | 3D1 | 1D3 | | | | | | 5D1 |
| | | 1D3 | 2D3 | | | | 8D3 | | | | | | | 14D3 |
| 14 | An Giang | 1D2 | | | 1D3 | | | | 1D3 | | | 1D3 | | 1D2 |
| | | 1D3 | | | | | | | | | | | | 4D3 |
| 15 | Bac Lieu | | | | | 1D3 | | | 1D3 | | | | | 2D3 |
| 16 | Ca Mau | | | | | 1D3 | | | | | | | | 1D3 |
| 17 | Kien Giang | 2D2 | | | | | | | | | | | | 2D2 |
| | | 8D3 | | | | | | | | | | | | 8D3 |
| Total | | 1D1 | 3D1 | | 3D3 | 5D3 | 5D1 | 2D1 | 6D1 | 2D3 | 4D2 | 4D3 | | 17D1 |
| | | 3D2 | 6D2 | | | | 2D2 | 6D2 | 19D3 | | 15D3 | 2D4 | | 21D2 |
| | | 12D3 | 6D3 | | | | 18D3 | 17D3 | | | 1D4 | | | 101D3 |
| | | 1D4 | | | | | | | | | | | | 4D4 |
| No./No. specimens | | 17/90 | 15/142 | 0/10 | 3/57 | 5/103 | 25/244 | 25/179 | 25/104 | 2/78 | 20/161 | 6/53 | 0/15 | 143/1,236 |

Dengue virus serotypes: D1 = DEN1; D2 = DEN2; D3 = DEN3; D4 = DEN4

Table 2. Specimens positive for dengue virus, by province, 1998

| Prov | Jan | Feb | Mar | Apr | May | Jun | Jul | Aug | Sep | Oct | Nov | Dec | Total & rate (%) |
|---|---|---|---|---|---|---|---|---|---|---|---|---|---|
| Lam Dong | | | | 0*/10 | 3/10 | | | | | | | | 3/20 (15) |
| Dong Nai | | | | | | 18/22 | 4/4 | | | 12/16 | | | 34/42 (80.95) |
| Br-V Tau | | | | | | 2/2 | 4/4 | | | | | | 6/6 (100) |
| HCMC | 9/38 | 35/82 | 6/23 | 20/48 | 14/21 | 38/54 | 78/117 | 71/79 | 40/83 | 63/162 | 24/54 | 16/66 | 414/827 (50.06) |
| Long An | | 0/38 | | | | 16/24 | | 6/7 | 2/3 | | | | 24/72 (33.33) |
| Tien Giang | | | | | 1/6 | 23/67 | | | 6/8 | 4/4 | 10/10 | 3/3 | 47/98 (47.96) |
| Ben Tre | | | | | 9/14 | | 25/32 | 16/21 | 12/26 | 3/3 | | | 65/96 (67.71) |
| Vinh Long | | 17/98 | | | | 41/57 | | | | | | | 58/155 (37.42) |
| Tra Vinh | | | | | | | 19/27 | 5/6 | 10/17 | 5/6 | | | 39/56 (69.64) |
| Dong Thap | 3/5 | | 1/3 | 1/5 | | | | | 3/4 | | | | 8/17 (47.06) |
| Can Tho | | | 2/3 | 1/2 | 11/17 | | | | | | | | 14/22 (63.64) |
| Soc Trang | | | | | | | 1/2 | 8/28 | | | | | 9/30 (30) |
| An Giang | 28/118 | 25/101 | 45/138 | 51/117 | 72/116 | 55/68 | 62/86 | 88/114 | 50/88 | 37/60 | 26/38 | 3/4 | 542/1,048 (51.72) |
| Ca Mau | | | | | 6/7 | 8/12 | 1/6 | | | 9/12 | 4/4 | | 28/41 (68.29) |
| Bac Lieu | | | | 1/2 | 3/11 | | | | | | | | 4/13 (30.77) |
| Kien Giang | 1/17 | 0/5 | | | | | 0/7 | | | 11/13 | 0/1 | 0/1 | 12/44 (27.27) |
| Total | 41/178 | 77/324 | 54/167 | 74/184 | 119/202 | 201/306 | 194/285 | 194/255 | 123/229 | 144/276 | 64/107 | 22/74 | 1,307/2,587 (50.52) |

*Number of positive specimens/total number of sera tested by IgM capture enzyme-linked immunosorbent assay (Mac-ELISA)

unknown origin. As a result, hospitals in these provinces were overwhelmed by patients, to the extent that the quality of treatment has been affected.

### Conclusions

During 1990-1998, dengue viruses were most often recovered in children 5 to 14 years of age (3). In the 1998 outbreak, more dengue viruses were isolated from adults (18.2%) than in the previous 4 years. Adults are not likely to have been exposed to the emerging DEN-3 virus.

From 1987 to 1998, the dengue virus serotypes in circulation changed (3). DEN-2 was responsible for the 1987 epidemic. From 1990 to 1995, DEN-1 predominated, but had decreased to 11.9% by 1998. DEN-2 accounted for 42.2% of the serotypes identified in 1997, but had decreased to 14.7% by 1998. The circulation of DEN-3 was the lowest during 1987-1994; increased to 29.5% by 1996, 42.2% by 1997, and 70.6% in 1998; and was the predominant serotype of the 1998 epidemic.

DEN-3 virus was first detected in 1987 only in Ho Chi Minh City, but by 1991 it was also identified in Tien Giang Province (7). In 1994 it appeared in Tien Giang and Soc Trang, in 1997 in four additional provinces, and by 1998 in 15 provinces. After a 5-year absence, DEN-4 virus was also detected in Dong Thap and Tra Vinh provinces in the Mekong Delta.

During a 1998 DHF epidemic affecting 19 provinces in southern Vietnam, 119,429 cases and 342 deaths were reported, for an increase of 152.4% and 151.8%, respectively, over 1997. It was the largest DHF epidemic in Vietnam since 1963. DEN-3, which began to emerge in southern Vietnam in 1994, was the serotype associated with the 1998 epidemic. The simultaneous emergence of DEN-4 should alert public health officials to the potential for outbreaks associated with that serotype. Virologic and serologic surveillance indicate that dengue is endemic in southern Vietnam and that the Dengue Control Program should be implemented in the interepidemic phase—in the first quarter of every year.

### Acknowledgments

We thank D.J. Gubler for providing the monoclonal antibodies used in this study.

Dr. Do Quang Ha is Head of the Arbovirus Laboratory, Pasteur Institute, Ho Chi Minh City, Vietnam. His research interests focus on arboviruses and the infectious diseases they cause.

### References

1. Do QH, Vu TQH, Huynh TKL, Cao MT. Dengue activity in southern Vietnam, 1995-1997. Journal of Medicine and Pharmacy Activity III 1998;special issue:259-63 (in Vietnamese).
2. Do QH, Nguyen KT, Dinh TH, Vu TQH, Nguyen TL, Vo DT, et al. Epidemic DHF in South Vietnam, 1987: epidemiological and virological studies. Dengue Newsletter (WHO) 1989;14:46-57.
3. Do QH, Vu TQH, Huynh TKL, Dinh QT, Deubel V. Dengue haemorrhagic fever in the south of Vietnam during 1975-1992 and its control strategy. Jap Trop Med 1994;36:187-201.
4. Do QH, Vu TQH, Huynh TKL, Pham KS. Situation of DHF in South Vietnam, 1991-1994. Dengue Bulletin WHO 1996;20:55-61.
5. Gubler DJ. Application of serotype-specific monoclonal antibodies for identification of dengue viruses. In: Yunker C, editor. Arboviruses in arthropod cells in vitro. Boca Raton: CRC Press; 1986. p. 3-14.
6. Kuno G, Gomez I, Gubler DJ. Detecting artificial anti-dengue IgM immune complexes using an enzyme-linked immunosorbent assay. Am J Trop Med Hyg 1987;36:153-9.
7. Do QH, Vu TQH, Huynh TKL, Cao MT. Virological surveillance of dengue haemorrhagic fever in southern Vietnam during 1987-1998. Vietnamese Journal of Preventive Medicine IX 1999;3:17-27 (in Vietnamese).
